# Progress Toward Polio Eradication — Worldwide, January 2016–March 2018

**DOI:** 10.15585/mmwr.mm6718a4

**Published:** 2018-05-11

**Authors:** Farrah Khan, S. Deblina Datta, Arshad Quddus, John F. Vertefeuille, Cara C. Burns, Jaume Jorba, Steven G.F. Wassilak

**Affiliations:** ^1^Global Immunization Division, Center for Global Health, CDC; ^2^Polio Eradication Department, World Health Organization, Geneva, Switzerland; ^3^Division of Viral Diseases, National Center for Immunization and Respiratory Diseases, CDC.

In 1988, when an estimated 350,000 cases of poliomyelitis occurred in 125 countries, the World Health Assembly resolved to eradicate polio globally. Transmission of wild poliovirus (WPV) continues uninterrupted in only three countries (Afghanistan, Nigeria, and Pakistan) (*1*), and among the three serotypes, WPV type 1 (WPV1) remains the only confirmed circulating type. This report describes global progress toward polio eradication during January 2016–March 2018, and updates previous reports (*2*). In 2017, 22 WPV1 cases were reported, a 41% decrease from the 37 WPV1 cases reported in 2016. As of April 24, 2018, eight WPV1 cases have been reported (seven in Afghanistan and one in Pakistan), compared with five cases during the same period in 2017. In Pakistan, continuing WPV1 transmission has been confirmed in multiple areas in 2018 by isolation from wastewater samples. In Nigeria, ongoing endemic WPV1 transmission was confirmed in 2016 (*3*); although WPV was not detected in 2017 or in 2018 to date, limitations in access for vaccination and surveillance in insurgent-held areas in northeastern Nigeria might permit continued undetected poliovirus transmission. Substantial progress toward polio eradication has continued in recent years; however, interruption of WPV transmission will require overcoming remaining challenges to reaching and vaccinating every missed child. Until poliovirus eradication is achieved, all countries must remain vigilant by maintaining high population immunity and sensitive poliovirus surveillance.

## Routine Poliovirus Vaccination Coverage

Among infants aged 1 year, the estimated global coverage with 3 doses of poliovirus vaccines (Pol3, mostly oral poliovirus vaccine [OPV]) through routine immunization services was 85% in 2016 (the most recent year for which data are available). World Health Organization (WHO)/United Nations Children’s Fund estimates for Pol3 coverage in 2016 were 73% in the African Region, 92% in the Region of the Americas, 80% in the Eastern Mediterranean Region, 94% in the European Region, 87% in the South-East Asia Region, and 95% in the Western Pacific Region, with heterogeneity in coverage among countries in all regions.* National Pol3 coverage with the third dose of OPV (OPV3) in the three countries with endemic WPV transmission in 2016 was 60% in Afghanistan, 72% in Pakistan, and 49% in Nigeria. OPV3 coverage is substantially lower in areas of WPV transmission, where children in high-risk mobile populations or areas of conflict are repeatedly missed (*4*,*5*). Rarely, in areas with low vaccination coverage, Sabin-like viruses can spread and revert to neurovirulence, resulting in outbreaks of disease caused by circulating vaccine-derived polioviruses (cVDPV). Approximately 90% of cVPDV cases reported since 2006 have been caused by type 2 (cVDPV2). In countries with recent cVDPV detections, Pol3 coverage was 74% in the Democratic Republic of the Congo (DRC), 48% in Syria, 47% in Somalia, and 83% in Laos (*6*). In these countries, OPV3 coverage was substantially lower in subnational areas with cVDPV emergence and transmission.

Following certification of the eradication of WPV type 2 (WPV2) in 2015, a global, synchronized withdrawal of trivalent OPV (tOPV, containing types 1, 2, and 3 live, attenuated polioviruses), and switch to bivalent OPV (bOPV, containing types 1 and 3 only), was completed by the end of April 2016 (*7*). Starting in 2015, injectable trivalent inactivated poliovirus vaccine (IPV) was introduced into routine immunization schedules in OPV-using countries, generally at 14 weeks of age. Some countries had to delay introduction of IPV until 2018 because of global shortages of the vaccine.

## Supplementary Immunization Activities

In 2016, 186 supplementary immunization activities (SIAs) were conducted in five WHO regions, during which approximately two billion total OPV and IPV doses were administered (Table 1), including 1,264,552,301 (63%) doses administered during national immunization days, 710,995,110 (36%) during subnational immunization days, and 17,603,036 (1%) doses during focused SIAs in areas of known or suspected poliovirus circulation (“mop-up” activities). In the event of cVDPV2 outbreaks, on advice of the monovalent OPV type 2 (mOPV2) Global Advisory Group, the WHO Director-General releases mOPV2 for outbreak response immunization. Of the administered doses, more than half (51%) were tOPV and approximately half (47%) were bOPV; an additional 1.4% were mOPV2, 0.05% were IPV plus bOPV, 0.2% were IPV alone, and 0.15% were fractional IPV (0.1 mL administered intradermally).

**TABLE 1:** Number of supplementary immunization activities (SIAs) conducted, and number of oral poliovirus vaccine (OPV) and inactivated poliovirus (IPV) doses administered, by World Health Organization (WHO) region — worldwide, 2016–2017

Year/SIAs/Vaccine doses administered	Region						
	Global	AFR	AMR	EMR	EUR	SEAR	WPR
**2016**							
**SIAs (no.)**	**186**	97	0	67	2	14	6
**Vaccine (no. of doses administered)**							
mOPV2	**28,357,599**	28,357,599	0	0	0	0	0
bOPV	**940,622,006**	274,197,570		397,909,506	54,880,271	206,507,773	7,126,886
tOPV	**1,017,074,205**	407,366,635	0	103,470,392	1,097,605	496,401,815	8,737,758
IPV	**3,293,021**	1,943,763		134,9258	0	0	0
IPV + bOPV	**904,050**	0	0	904,050	0	0	0
fIPV	**2,899,566**	0	0	252,354	0	2,647,212	0
Total doses	**1,993,150,447**	711,865,567	0	503,885,560	55,977,876	705,556,800	15,864,644
**2017**							
**SIAs (no.)**	**172**	82	0	79	2	8	1
**Vaccine (no. of doses administered)**							
mOPV2	**70,356,186**	65,067,196	0	5,288,990	0	0	0
bOPV	**1,705,913,274**	519,920,180	0	488,368,342	389,314	696,180,796	1,054,642
tOPV	**0**	0	0	0	0	0	0
IPV	**3,522,237**	558,897	0	2,963,340	0	0	0
IPV + bOPV	**8,920,134**		0	8,920,134	0	0	0
fIPV	**0**	0	0	0	0	0	0
Total doses	**1,788,711,831**	585,546,273	0	505,540,806	389,314	696,180,796	1,054,642

**Abbreviations:** AFR = African Region, AMR = Region of the Americas; bOPV2 = bivalent oral poliovirus, types 1 and 3; EMR = Eastern Mediterranean Region; EUR = European Region; fIPV = fractional dose inactivated poliovirus vaccine (one fifth of a 0.5 mL intramuscular dose, given intradermally); IPV = inactivated poliovirus vaccine; mOPV2 = monovalent oral poliovirus, type 2; SEAR = South-East Asia Region; tOPV2 = trivalent oral poliovirus, types 1, 2, 3; WPR = Western Pacific Region.

In 2017, 172 SIAs were conducted in five WHO regions, during which approximately 1.79 billion total OPV and IPV doses were administered, including 1,110,923,756 (62%) doses administered during national immunization days, 672,091,158 (38%) during subnational immunization days, and 5,696,917 (0.3%) during mop-up activities. Of the administered doses, 95% were bOPV, 3.9% were mOPV2, 0.5% were IPV plus bOPV, and 0.2% were IPV alone.

## Poliovirus Surveillance

Surveillance for acute flaccid paralysis (AFP) is the means of detecting polio cases caused by WPV or cVDPV, confirmed by stool specimen testing through the Global Polio Laboratory Network. The performance of AFP surveillance is assessed through two main indicators: sensitivity and completeness of case investigation. An annual nonpolio AFP rate of ≥1 case per 100,000 population aged <15 years for countries in the WHO regions certified as poliofree, or ≥2 for all other countries is considered sufficiently sensitive to detect a case of polio, should it occur. Case investigation is considered to be sufficiently complete if at least 80% of reported AFP cases have adequate stool specimens collected (i.e., two stool specimens collected ≥24 hours apart, within 14 days of paralysis onset, with arrival at a WHO-accredited laboratory in good condition). In 2016, among the four countries reporting polio cases, three (Afghanistan, Nigeria, Pakistan) met both performance indicators and one (Laos) did not. Among the five countries reporting polio cases in 2017, four (Afghanistan, DRC, Nigeria, Pakistan) met both performance indicators and one (Syria) did not. Although Nigeria and DRC meet AFP surveillance indicators nationally and subnationally in most provinces, both countries are affected by substantial issues in population accessibility and other impediments to AFP surveillance (*1*). AFP surveillance has been supplemented by environmental surveillance through testing of sewage in many countries, including poliofree countries as well as those with endemic transmission (*1*).

## Reported Poliovirus Cases

**Countries reporting WPV cases.** In 2016, 37 WPV cases were detected (Figure): 13 (35%) in Afghanistan, 20 (54%) in Pakistan, and four (11%) in Nigeria. In 2017, 22 WPV cases were identified: 14 (64%) in Afghanistan and eight (36%) in Pakistan. No WPV cases have been identified in countries outside of Afghanistan, Nigeria, and Pakistan since 2014. During January 1–March 30, 2018, as of April 24, the low poliovirus transmission season, eight WPV1 cases were reported (seven in Afghanistan; one in Pakistan) (Figure) (Table 2).

**FIGURE:**
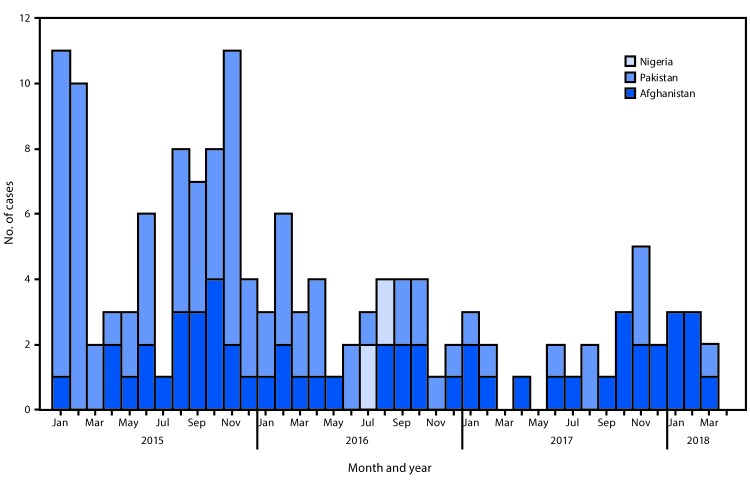
Number of cases of wild poliovirus, by month of onset — worldwide, January 2015–March 2018* * Data as of April 24, 2018.

**TABLE 2:** Number of reported polio cases, by country — Worldwide, January 1, 2016–March 30, 2018*

Classification/Country	2016 (Jan 1–Dec 31)		2017 (Jan 1–Dec 31)		2017 (Jan 1–Mar 30)		2018 (Jan 1–Mar 30)	
	WPV	cVDPV	WPV	cVDPV	WPV	cVDPV	WPV	cVDPV
**Countries with endemic polio**								
Afghanistan	13	0	14	0	3	0	7	0
Pakistan	20	1	8	0	2	0	1	0
Nigeria	4	1	0	0	0	0	0	0
**Total cases in endemic countries**	**37**	**2**	**22**	**0**	**5**	**0**	**8**	**0**
**Other countries with reported cVDPV cases**								
Laos	0	3	0	0	0	0	0	0
Democratic Republic of the Congo	0	0	0	22	0	0	0	3
Syria	0	0	0	74	0	0	0	0
**Total cases in other countries**	**0**	**3**	**0**	**96**	**0**	**0**	**0**	**3**
**Total paralytic polio cases**	**37**	**5**	**22**	**96**	**5**	**0**	**8**	**3**

**Abbreviations:** cVDPV = circulating vaccine-derived poliovirus; WPV = wild poliovirus.

* Data as of April 24, 2018.

Afghanistan reported 13 WPV1 cases in four districts in 2016, compared with 14 WPV1 cases in nine districts in 2017 (7.7% increase). In 2016, 54% of WPV1 cases in Afghanistan were reported from Paktika province in the southeastern region. In 2017, 50% of WPV1 cases were reported from Kandahar province in the southern region. During January 1–March 30, 2018, seven WPV1 cases were detected (four in Kandahar province, one in Nangahar province, and two in Kunar province; the latter two provinces are in the eastern region), compared with three WPV1 cases detected during the same period in 2017.

Pakistan reported a 60% decrease in the number of WPV1 cases, from 20 cases in four districts in 2016 to eight cases in seven districts in 2017. During January 1–March 30, 2018, one WPV1 case was reported (in Balochistan province), compared with two reported during the same period in 2017. WPV1 continues to be isolated from environmental surveillance sites in five provinces of the country (Balochistan, Islamabad, Khyber Pakhtunkhwa, Punjab, and Sindh).

Nigeria reported four WPV1 cases in 2016. No WPV1 cases were reported in 2017 and none to date in 2018.

**Countries reporting cVDPV cases and isolations.** In 2016, five cVDPV cases were reported from three countries (*8*). In Laos, an outbreak that began with eight cVDPV type 1 cases in 2015 continued into 2016 with three additional cases reported. One cVDVPV2 case was reported in 2016 in Nigeria and another in Pakistan. In 2017, a total of 96 cVDPV2 cases were reported, including 74 cases from Syria (most recent case in September 2017) and 22 from DRC. The outbreak in DRC has continued into 2018, with four cases to date, as of April 24, 2018 (the most recent case occurring in February) (*9*). Isolation of cVDVP2 from environmental samples in Mogadishu, Somalia, in late 2017 and early 2018, and related cVDPV2 from environmental samples in Nairobi, Kenya, in early 2018, has confirmed long-term cVDPV2 transmission, in a broad area, although no associated polio cases have been detected to date. cVDPV type 3 has been isolated in Mogadishu from sewage samples collected in March 2018, again, with no associated polio cases having been detected to date. In Nigeria, cVDPV2 has been recently detected by environmental surveillance in two states in early 2018; no associated polio cases having been detected to date. Response immunization is underway or planned for all these cVDPV cases and isolations.

## Discussion

Although substantial progress was made toward polio eradication during 2016–2017, challenges remain in the countries with endemic transmission. Continued circulation of WPV1 has been confirmed in Afghanistan and Pakistan in the 2018 low WPV season, and it remains uncertain if WPV circulation has been interrupted in Nigeria (*3*).

The number of WPV cases in Afghanistan declined from 2015 to 2016, but the decrease did not continue in 2017. Although negotiations to obtain local access are constantly being undertaken, the number of children who were inaccessible to vaccination in the south and east because of insecurity increased during 2017 (*5*). In Pakistan, a decline in WPV1 cases since 2014 continued during 2016 and 2017. The detection of WPV in environmental surveillance samples in the absence of WPV-positive AFP cases in several provinces might indicate either surveillance gaps or waning in the intensity of transmission. Intensified SIA schedules and efforts to reach previously unvaccinated children, along with expansion of community-based initiatives employing local permanent vaccinators and ensuring worker safety have helped reduce the number of WPV cases. Large-scale movement of high-risk populations across Pakistan’s border with Afghanistan in both directions continues to pose a challenge to interrupting WPV transmission, and crossborder collaborative vaccination efforts made in 2017 are being enhanced in 2018 (*4*).

In Nigeria, WPV1 circulation went undetected from mid-2014 to mid-2016, and the discovery of both endemic WPV1 and long-standing cVDVP2 transmission in 2016 in Borno State illuminated gaps in surveillance. Continued inaccessibility of insurgent-held areas hinders both immunization and surveillance efforts (*3*). Enhancement of initiatives for collaborating with the military to reach currently unvaccinated children will be helpful in ensuring interruption of WPV transmission. In the other countries of the Lake Chad basin bordering Borno State (Cameroon, Chad, and Niger), problems with inaccessibility related to insecurity and a large number of difficult-to-access islands have been addressed through progressive improvements in microplanning and implementation of SIAs, but uncertainties remain regarding SIA quality and success in interrupting undetected WPV transmission.

Global WPV2 eradication was certified in 2015 after no detection since 1999 (*2*). WPV type 3 has not been detected since 2012 (*2*). A minimum of 3 years of sensitive AFP surveillance without detection of WPV is required to certify a WHO region as being poliofree (*10*). Four of six WHO regions (the Region of the Americas, European, South-East Asia, and Western Pacific regions) have been certified free of indigenous WPV. Improvements in AFP surveillance performance in critical subnational areas are required to achieve poliofree certification of the African and Eastern Mediterranean regions.

Because efforts to increase immunity to poliovirus type 2 before the global tOPV to bOPV switch did not reach all persistently unvaccinated children in hard-to-reach areas, some cVDPV2 emergences have been detected following the switch. Reaching all children for vaccination in areas with cVDPV2 transmission is also an ongoing challenge. 

Although progress toward global polio eradication has continued, challenges in identifying and vaccinating every missed child remain. Much of the recent progress reaching previously missed children has been associated with recruitment of trusted community volunteers who are invested in their locality for vaccination and surveillance efforts. Intensification of efforts to improve the quality of immunization and surveillance activities and to develop additional innovations in addressing persisting challenges is necessary. Until poliovirus eradication is achieved, all countries must remain vigilant by maintaining high population immunity and sensitive poliovirus surveillance.

**Summary**

**What is already known about this topic?**

Transmission of wild poliovirus type 1 (WPV1) has not been interrupted in Afghanistan, Nigeria, and Pakistan. A global, synchronized switch to bivalent oral poliovirus vaccine (bOPV, types 1 and 3 only) was completed in April 2016.

**What is added by this report?**

Compared with 2016, the number of WPV1 cases overall decreased in 2017. Some transmission of circulating vaccine-derived poliovirus type 2 (cVDPV2) has been identified more than 1 year following the switch to bOPV in 2016.

**What are the implications for public health practice?**

Interruption of transmission of WPV1 and of cVDPV2 will require addressing persistent challenges to vaccinating every missed child. Until poliovirus eradication is achieved, all countries must maintain high population immunity and sensitive poliovirus surveillance.

## Footnotes

* http://apps.who.int/immunization_monitoring/globalsummary/timeseries/tswucoveragepol3.html.
